# The challenge of eco-generativity. Embracing a positive mindset beyond eco-anxiety: a research agenda

**DOI:** 10.3389/fpsyg.2024.1173303

**Published:** 2024-04-05

**Authors:** Annamaria Di Fabio, Andrea Svicher

**Affiliations:** ^1^Department of Education, Languages, Intercultures, Literatures and Psychology (Psychology Section), University of Florence, Florence, Italy; ^2^THE-Tuscany Health Ecosystem NextGeneration UE-NRRP, Department of Education, Languages, Intercultures, Literatures and Psychology (Psychology Section), University of Florence, Florence, Italy

**Keywords:** eco-generativity, ecological generativity, hope, eco-anxiety, climate change, sustainability, sustainable development, psychology of sustainability and sustainable development

## Abstract

Climate change has emerged as a tough challenge affecting the world’s society and economy in the twenty-first century. Furthermore, it has been determined that global warming and climate change have detrimental effects on human health both physical and psychological. In this framework, eco-anxiety has emerged as a new construct to assess the distress in relation to climate change and its effects. In the current article, after a study of the literature regarding both eco-anxiety and generativity related to environmental issues, in the search for a healthy response to eco-anxiety, we propose the construct of eco-generativity as a sustainable development-related concept for the health of planet earth and people in the present and in the future. Accordingly, we explore the definitions of generativity in relation to the ecological environment, examining the development of the concept in accordance with the most recent research. Subsequently, according to the lens of psychology of sustainability and sustainable development, we propose key elements of eco-generativity in terms of construct and measures. Finally, a research agenda for future research and intervention on eco-generativity is provided.

## Introduction

Nowadays, dealing with the global climate crisis is the most crucial issue for 21^st^ century societies and economies, as well as a major concern for environmental and human health (e.g., Morrison et al., 2022; Heeren and Asmundson, 2023). Climate change is shift in temperature and weather variability, including a rise in the frequency and severity of extreme environmental events (Mariappan et al., 2022). Downstream implications of climate change impact the environment (e.g., forest degradation, desertification, forest fires, lack of freshwater supplies, decreasing ecosystem functionality and biodiversity) negatively impacting economic growth and human health (Watts et al., 2021). The health of populations is damaged in several ways (World Health Organization, 2021; Nadeau et al., 2022), with a widespread magnitude of negative psychological effects (Palinkas and Wong, 2020). Global warming and climate change have been recognized to “deleteriously affect many aspects of planetary and human health.” (Nadeau et al., 2022, p. 1087). As a result, resilience to climate change is a keyword that firmly informs sustainability research (e.g., Satterthwaite et al., 2020).

The latest released reports by the United Nations Intergovernmental Panel on Climate Change (IPCC) have highlighted that the goal to limit global warming could be attained if climate neutrality (i.e., worldwide zero carbon emissions) was attained between 2030 and 2050 (Intergovernmental Panel on Climate Change, 2022). Despite this, global temperatures will continue to rise until 2050, albeit many climate preventive actions have been planned (Intergovernmental Panel on Climate Change, 2022). Thus, concerns about a sustainable future for life on earth are becoming one of the most compelling worldwide scientific, political, and informative debates (Cianconi et al., 2023). In turn, a widespread emerging research line in applied psychology has examined anxiety, worry, and concerns that individual has experienced in facing the challenges of climate change (e.g., Boluda-Verdú et al., 2022).

## Eco-anxiety

In this scenario, a growing body of literature have highlighted an emergent psychological phenomenon concerning the climate crisis, labeled *“eco-anxiety”* (Boluda-Verdú et al., 2022). Eco-anxiety is defined as “*a chronic fear of environmental doom*” characterized by worries regarding the inadequacy of climate actions and adverse effects of warming crisis (Clayton et al., 2017, p. 68). Other labels used by researchers interchangeably are climate anxiety (Boyd et al., 2023), climate change worry (Stewart, 2021), environmental distress (Higginbotham et al., 2006), ecological stress (Helm et al., 2018), and ecological grief (Cunsolo and Ellis, 2018). Data from a cross-national survey on adolescents revealed that 59% of them were very or extremely worried about climate change and more than 45% had impairment of everyday functioning (e.g., affecting ability to work and/or socialize) due to eco-anxiety (Hickman et al., 2021). Similar results have been observed in adults (Clayton and Karazsia, 2020) and observed around the globe (e.g., Gibson et al., 2020; Hajek and König, 2022; Heeren et al., 2022; Massazza et al., 2022; Tam et al., 2023).

Starting from this growing phenomenon, scholars have developed measurement tools to investigate eco-anxiety. In this light, the most widely used tool (Boluda-Verdú et al., 2022) is the Climate Change Anxiety Scale (CCAS) (Clayton and Karazsia, 2020), a 22-item scale measuring difficulties caused by the changing climate on four factors with adequate reliability: difficulties both on a cognitive level and on an emotional level, impairments on a functional level, personal experience of climate change, behavioral engagement. A brief version of the CCAS, enclosing a 13-item reliable two-factor structure, was advanced by Mouguiama-Daouda et al. (2022). Stewart (2021) proposed the Climate Change Worry Scale (CCWS), a 10-item unidimensional scale measuring worry (persistent, repetitive, and uncontrolled thoughts) about climate change, concerns on future changes that climate may bring, and dysfunctional responses to worries, showing a one-factor solution with good psychometric properties (Stewart, 2021). Other scales that can be found in the literature were all created *ad hoc* for research, with psychometric properties partially demonstrated and needing further study: Climate Change distress scale (Searle and Gow, 2010); Habit Index of Negative Thinking adaptation (Verplanken et al., 2020); Negative climate-related emotions (Ogunbode et al., 2021); Eco-emotion scale (Stanley et al., 2021). Even though eco-anxiety has been studied and operationalized in the most recent literature, it represents an ongoing challenge for sustainable development and sustainability research (e.g., Wang et al., 2023). Furthermore, researchers could embrace also different lens to study the psychological perspective of individuals that are living such adversities. For example, embracing a positive-oriented perspective, also in terms of sustainable development (Seligman and Csikszentmihalyi, 2000; Di Fabio, 2017; Di Fabio and Rosen, 2020), focusing on the psychological resources that individuals have at their disposal to cope with climate change anxiety. This perspective could open new research trajectories applying positive psychological resources to promote sustainability and sustainability-related processes for the health and well-being of individual/s and the environment/s.

## The psychology of sustainability and sustainable development

During the last 10 years, sustainability science has emerged as a novel academic discipline that addresses the challenges related to sustainable development through transdisciplinary lens, integrating natural, applied, and social sciences, as well as humanities (Rosen, 2009, 2017; Dincer and Rosen, 2013). Sustainable development is traditionally focused on strategies that could preserve the planet’s heart and human society from the ever-increasing degradation of environmental resources, promoting the protection of the environment and its ecosystem in the future (Rosen, 2017). Nowadays, sustainability science (Rosen, 2009, 2017; Dincer and Rosen, 2013) participates directly and contributes to United Nations (UN) 17 Sustainable Development Goals (SDGs), bringing its contribution to overcome the major challenges including environmental degradation, climate change, and human well-being (United Nations, 2022). More recently, a novel research area has stemmed from the sustainability science realm, namely the *psychology of sustainability and sustainable development* (Di Fabio, 2017; Di Fabio and Rosen, 2018, 2020): it proposes to integrate psychological lens in the advancement of sustainability and sustainable development. Additionally, the psychology of sustainability and sustainable development offers a psychological outlook accounting for many environments (Di Fabio and Rosen, 2018, 2020) and their interrelationships, starting from natural environment and its ecosystem including other environments such as personal/individual, social, organizational, community, digital, cross-cultural… up to global environment. Moreover, the psychology of sustainability and sustainable development is aimed at supporting the principle that sustainable processes have to be handled both by adhering to ever-decreasing supply of resources and even by regenerating resources following a positive-oriented approach (Di Fabio, 2017, 2022).

Therefore, according to this perspective, it sounds useful to switch views in studying the climate crisis concerns. This switch pertains to detecting positive variables that could regenerate psychological resources, and facilitate adaptive processes related to sustainability and sustainable development. In turn, focusing on positive psychological variables could help individuals in overcoming the limitations of focusing only on stagnation and/or negative affective states. As a result, embracing a positive-oriented approach could be not only a strategy to help individuals overcome the current environmental concerns but also to expand the field of sustainability, exploring psychological processes and resources able to open more profitable opportunities based on enhancing well-being and health of individual/s and environment/s. Eco-generativity is promising in this perspective as a viaticum to build a constructive proposal more generally, and it may be so in relation to eco-anxiety as well.

## Eco-generativity

In recent years, researchers have extensively studied the construct of generativity (Thomas and Tee, 2022) also outside the traditional boundaries of personality research (e.g., Doerwald et al., 2021; Wiktorowicz et al., 2022). However, despite environmental and ecological issues constitute a major global concern, only a handful of studies also investigated ecological generativity (e.g., Schoklitsch and Baumann, 2011; Alisat et al., 2014).

*Eco-generativity* is a concept that, on one side follows the evolution of the construct of generativity, which was first provided by Erikson in 1963; on the other side it extends the idea of generativity to the environment and the natural world and deals with passing the environment to subsequent generations, assisting the future of humankind (Schoklitsch and Baumann, 2011).

Focusing on the construct of generativity in the scientific literature, also reporting relevant moments of enrichment of the perspective, the starting point to consider is the contribution of Erikson (1963, 1968, 1974, 1980, 1982, 1986). According to Eriksonian psychosocial stages and tasks, generativity is the seventh of eight personality development phases opposed to stagnation. Generativity is defined by Erikson as *“the establishment, the guidance, and the enrichment of the living generation and the world it inherits”* (Erikson, 1974, p. 123), and it is in relation to adults capable to define a perspective of being engaged in long-lasting affective interpersonal partnerships, and able to dedicate themselves to the next generations, nourishing and guiding them (Erikson, 1963, 1968). Furthermore, generativity deals with the capacity to provide a creation of the adult self, as a kid, a book, an idea, or a piece of knowledge that is deliberately and unselfishly shared with others and made to leave something behind, encouraging generational continuity (Erikson, 1963, 1968). Afterwards, scholars have gone beyond the notion of a “*generativity stage*,” emphasizing the presence of several facets of generativity, capable to be present in the individuals’ personality from early to late adulthood (McAdams et al., 1993). McAdams et al. (1986) conceive generativity as a two-step process, containing elements of caring for subsequent generations and agentic aspects of leaving an entail of self beyond death. McAdams and de St. Aubin (1992) proposed the *theory of generativity* to illustrate generativity as a multidimensional personality construct composed of seven facets that could be exhibited in early, middle, or elder adulthood: (1) cultural demand; (2) inner desire; (3) concern for the next generation; (4) belief in the goodness of the human species; (5) generative commitment; (6) generative action; (7) narration of generativity (McAdams and de St. Aubin, 1992). They are individually arranged, elicited by psychosocial demands (e.g., environmental, biological, psychological, social, cultural) and addressed to the goal of nourishing the following generation.

From another point of view, Kotre (1984) differentiated four distinct forms of generativity, removing any form of restrictions based on age or societal roles: biological (e.g., nursing children), parental (e.g., providing food, clothes, love, and discipline), technical (accomplished by teachers transmitting skills), and cultural (teachers who transmit not only skills but their meanings) (Kotre, 1984).

Another group of scholars (Bradley, 1997; Bradley and Marcia, 1998; Morselli, 2013; Morselli and Passini, 2015) focuses on the links between future time perspective (Zimbardo and Boyd, 1999) and generativity, projecting themselves into the future being aware of future consequences nurtured by social responsibility. Following these premises, Morselli and Passini (2015) proposed the concept of *social generativity* describing an inclusive attitude towards society, not only a set of purposes fueled by personal and instrumental goals (Marcia, 2010), but rather the responsibility for successive generations being involved in actions in the present in favor of the community’s future. Lastly, a recent systematic review of literature (Doerwald et al., 2021) has underlined that generativity has a valuable role in the workplace and it was associated with a large array of work-related outcomes and well-being, suggesting including it in the area of the positive psychological resources.

Currently, an increasingly interesting space is emerging in relation to the application of generativity to environmental challenges. In the literature McAdams and de St. Aubin (1992) included environmental issues in generative concerns as motivational sources for pushing individuals towards generative actions but they did not further expand this concept. Schoklitsch and Baumann (2011) provided the first overlook on ecological generativity although considering it as the third factor of a broader measurement model together with Kotre’s (1984) four forms of generativity. Alisat et al. (2014) explored relationships between generativity and individual response to environmental issues observing that generativity was positively associated with environmental identity, environmental narratives, and strong feelings of connection with nature. However, the aforementioned authors did not further expand the concept in terms of ecological generativity. The lack of clear concepts and measures associated with ecological generativity, also without a multi-dimensional operationalization of the construct, may have limited the research and the chance to deeply explore the relationship between ecological generativity, positive psychological variables, well-being, and sustainable-related variables, highlining an open issue regarding the measure of the construct.

## Measuring eco-generativity

With the present contribution, we advance new coordinates to expand the scenario in terms of eco-generativity, enriching the perspective in line with the principles of the psychology of sustainability and sustainable development (Di Fabio, 2017; Di Fabio and Rosen, 2018, 2020).

By reviewing the materials available in the literature to measure aspects linked to this construct, three empirically validated measures are available in relation to different aspects involved. The first one is the ecological generativity factor, included in Gen-Current (current generative concerns) and Gen-Life (lifetime generative concerns) questionnaires (Schoklitsch and Baumann, 2011), two 29-item mirror measures composed of four factors: technical, cultural, social, and ecological generativity. The Ecological generativity factor covers the following concerns: (1) Use the energy wisely; (2) Leave a clean environment behind; (3) Live ecology-minded; (4) Keep waste to a minimum; (5) Purchase organic food; (6) Take care of animals; (7) Aid social institutions generativity (Schoklitsch and Baumann, 2011).

Another measure linked to another facet of Eco-generativity is the Social Generativity Scale (Morselli and Passini, 2015), covering aspects of eco-generativity since social generativity encloses concerns about future generations and the impact of individual behaviors on the community’s future. Social Generativity Scale showed a reliable unidimensional factor structure being composed of six items about: (1) undertaking initiatives to maintain the planet for the benefit of the next generation; (2) having sense of responsibility to support the neighborhood in which individual lives; (3) donating a portion of everyday commodities supporting the growth of future peoples; (4) being committed to ensuring the wealth of succeeding generations; (5) dedicating oneself to activities that survive even after individuals pass away; (6) assisting individuals in personal improvement (Morselli and Passini, 2015).

The third measure linked to another facet of Eco-generativity is the revised Environmental Identity scale (IED-R) (Clayton et al., 2021). Eco-generativity encloses features of environmental identity since it is composed of identity concerns associated with the natural world (Alisat et al., 2014). Furthermore, generativity, environmental concerns, and identity are strongly associated and mutually influential (Milfont and Sibley, 2011; Matsuba et al., 2012). The IED-R (Clayton et al., 2021) is a 14-item scale assessing cognitive, behavioral, and emotional aspects of how individuals view their relationship with nature, developed from the original 24-item environmental identity scale (Clayton, 2003), showing superior psychometric prosperities, cross-cultural validity, and adequate factor structure. It covers aspects associated with considering oneself a part of nature, devoting resources to protecting the context of nature, living a sustainable lifestyle, and feeling relaxed in nature (Clayton et al., 2021). Thus, these three measures could constitute a starting point to promote research examining facets of eco-generativity and its relationship with psychological processes associated with sustainability and positive psychological variables.

## Advancing a sustainable development-related concept of eco-generativity

As a first step, we defined the construct of eco-generativity as a specific form of generativity. This hallmark could be represented on the one hand by ecological concerns, following McAdams and de St. Aubin (1992). On the other hand, we have to include the social dimension of generativity (Morselli and Passini, 2015) since eco-generativity encapsulates a future-time perspective of care for environment and people, caring of the natural world as a fully livable and healthy environment for future generations, also including engagement in activism to preserve the environment.

These two faces of the current construct of eco-generativity appear consistent with the psychosocial lens of generativity theory, being ecological concerns activated by cultural demands and contingent aspects of the everyday life of the XXI century.

A second step in defining the eco-generativity construct requires a reflection on two concepts strictly related to eco-generativity. Environmental identity, as well as belief in the goodness of the human species to activate the passage from generativity concerns to generative commitment, actions, and narratives (McAdams and de St. Aubin, 1992) seem critical elements also in terms of positive motivational aspects of confidence and of success in the future. They could be necessary for having and renovating psychological domains (cognitive, emotional, and behavioral) (Matsuba et al., 2012; Alisat et al., 2014) in favor of eco-generativity. The environmental identity could be well reflected by the Clayton et al.’s (2021) construct assessed via the trustworthy and psychometrically sound 14 items of IED-R, as previously introduced. Regarding the belief in the goodness of the human species being a non-operationalized construct, it could be covered by the empirical construct of Hope (Snyder et al., 1991). The Hope Scale (Snyder et al., 1991), is a 12-item questionnaire with a reliable two-factor structure: Agency as a feeling of accomplishment in achieving objectives in the past, present, and future; Pathways as the ability to create effective strategies to achieve objectives (Snyder et al., 1991).

In general, a sustainable development-related concept of eco-generativity could encompass two core features encapsulated in the constructs of ecological generativity (Schoklitsch and Baumann, 2011) and social generativity (Morselli and Passini, 2015), addressing the major eco-generativity concerns and two additional features represented by environmental identity (Clayton et al., 2021) and hope (Snyder et al., 1991).

Another important step asks for the right placement of generativity in the hierarchy levels of personality-related domains, conceptualizing it as a personality-related domain that is separated, even though associated with personality traits. In the literature, recent studies (Navarro-Prados et al., 2018; Serrat et al., 2018; Millová et al., 2021) underline these relationships. Furthermore, Doerwald et al. (2021) conducted a meta-analysis where generativity emerges as a positive psychological resource positively associated with work-related outcomes. According to that, eco-generativity could be conceptualized in a positive strength-based perspective (Di Fabio and Saklofske, 2021) as a positive psychological resource implementable via specific training. Nevertheless, all the advanced steps require empirical investigation to be satisfactorily explored; therefore a research agenda for eco-generativity needs to be drafted for promoting the study of its relationship with health, wellbeing, and positive psychological aspects related to the natural world, environment, and sustainability.

## An eco-generativity research agenda

To cope with the challenge of the global climate crisis and several environmental issues, a new construct in the generativity framework is proposed: eco-generativity. Stemming from a generativity perspective (e.g., McAdams and de St. Aubin, 1992) it could be composed of four constituents: ecological generativity (Schoklitsch and Baumann, 2011), social generativity (Morselli and Passini, 2015), environmental identity (Clayton et al., 2021) and hope (Snyder et al., 1991). To be effectively introduced into the research landscape, the construct of eco-generativity should be investigated through a research agenda. It could enclose five points.

- An in-depth study of the factor structure of the construct implementing psychometric analytic strategies.
- An investigation on antecedents and outcomes of eco-generativity to better clarify the role of environmental identity and hope.
- An examination of relationships among eco-generativity and relevant personality construct and/or intrinsically related, such as personality traits (Costa and McCrae, 2008), emotional intelligence (Petrides and Furnham, 2001), and perfectionism (Hewitt et al., 1991; Feher et al., 2020).
- An analysis of relationships between eco-generativity and positive psychological resources, such as empathy (Davis, 1980), compassion (Goetz et al., 2010), life satisfaction (Diener et al., 1985), meaning in life (Morgan and Farsides, 2009), flourishing (Diener et al., 2010), humor (Martin et al., 2003; Ruch et al., 2018).
- A reflection on the value to introduce eco-generativity in the domain of positive psychological resources, positive strength-based perspective (Di Fabio and Saklofske, 2021), as well as in a positive preventive perspective (Di Fabio and Kenny, 2019).

Overall, the purpose of the current agenda is to promote the study of eco-generativity (Di Fabio and Svicher, 2023a,b) as a promising construct in the psychology of sustainability and sustainable development area, favoring its understanding and development to take a constructive perspective on coping with concerns associated with climate and environmental issues.

## Conclusion

Global climate change and its linked impacts, such as global warming and acute and extreme weather events, are all well-documented hazards to human health and well-being (Centers for Disease Control and Prevention, 2022). Moreover, these impacts are widespread and cumulative, burdening the psychological well-being of humanity (World Health Organization, 2021). In such a scenario, the new concept of eco-anxiety has advanced, enclosing worry for the environment and severe individual impairments (Clayton and Karazsia, 2020). Differently, eco-generativity entails caring for future environments and generations, acting from the present, as well as fostering environmental identity and hope. In this light, eco-generativity could also be a healthy response to the insecurity and stagnation arising from eco-anxiety, reinforcing the psychology of sustainability and sustainable development in helping individuals to cope positively with environmental challenges.

Through this approach, eco generativity could represent a promising candidate to enrich the study of the relationships between positive psychological resources and psychological coordinates of sustainable development (e.g., Di Fabio and Rosen, 2018, 2020). Accordingly, sustainable development from a psychological point of view is also related to promoting the health and wellbeing of individual/s and their environment/s, fostering positive connections between people and the natural world to support sustainability efforts and well-being. Future research perspectives could investigate the relationship between eco-generativity, well-being, health, and eco-health variables. Thus, eco-generativity could be a new positive-oriented variable for fostering psychological strengths, assisting individuals in their well-being as well as in taking care of the sustainable development of planet earth.

## Data availability statement

The original contributions presented in the study are included in the article/supplementary material, further inquiries can be directed to the corresponding author/s.

## Author contributions

AS wrote the first draft of the manuscript. ADF conceptualized the manuscript, supervised and tutored AS, and reviewed, edited, and wrote the final draft of the manuscript. All authors contributed to the article and approved the submitted version.
